# Association between Alcohol Consumption and Pancreatic Cancer Risk: A Case-Control Study

**DOI:** 10.1371/journal.pone.0124489

**Published:** 2015-04-09

**Authors:** Farah Rahman, Michelle Cotterchio, Sean P. Cleary, Steven Gallinger

**Affiliations:** 1 Dalla Lana School of Public Health, University of Toronto, Toronto, Ontario, Canada; 2 Prevention and Cancer Control, Cancer Care Ontario, Toronto, Ontario, Canada; 3 Department of Surgery, University Health Network, Toronto, Ontario, Canada; 4 Prosserman Centre for Health Research, Lunenfeld-Tanenbaum Research Institute, Toronto, Ontario, Canada; 5 Lunenfeld-Tanenbaum Research Institute, Mount Sinai Hospital, Toronto, Ontario, Canada; 6 Division of General Surgery, Toronto General Hospital, Toronto, Ontario, Canada; Mayo Clinic, UNITED STATES

## Abstract

**Purpose:**

Evidence is inconsistent regarding alcohol and pancreatic cancer risk, although heavy drinking may increase risk.

**Methods:**

A population-based case-control study was conducted using 345 pancreas cancer cases diagnosed 2011–2012 and 1,285 frequency-matched controls from Ontario, Canada. Logistic regression was used to evaluate alcohol consumption and pancreatic cancer risk; data was also stratified by sex and smoking status to assess interaction.

**Results:**

Alcohol consumption was not associated with pancreatic cancer risk (age-adjusted odds ratio=0.78, 95% CI: 0.58, 1.05 for 1 - 3 drinks/week; age-adjusted odds ratio=0.86, 95% CI: 0.63, 1.17 for 4 - 20 drinks/week), however there was a non-significant increased risk for heavy drinkers consuming ≥21 drinks/week (age-adjusted odds ratio=1.35, 95% CI: 0.81, 2.27). Cigarette smoking modified the alcohol-cancer relationship; among current smokers, heavy alcohol consumption was associated with a significantly increased pancreatic cancer risk (age-adjusted odds ratio=4.04, 95% CI: 1.58, 10.37), whereas this significant association with heavy drinking was not observed among non-smokers (age-adjusted odds ratio=2.01, 95% CI: 0.50, 8.18). Furthermore, light – moderate alcohol intake was associated with increased pancreas cancer risk among current smokers.

**Conclusions:**

While alcohol was not significantly associated with pancreatic cancer risk, smoking status modified this relationship such that among current smokers, alcohol intake was associated with a greater than two-fold increased risk of pancreatic cancer. The results should be interpreted with caution due to small sample sizes within subgroups and correction for multiple comparisons should be considered. These findings should be replicated in larger studies where more precise estimates of risk can be obtained.

## Introduction

Pancreatic cancer is one of the most lethal of all cancers [1]. In 2013, approximately 4,700 Canadians were diagnosed with pancreatic cancer and 4,300 died from it, with a 5-year survival rate of less than 10% [1]. The poor survival rates are thought to be due to lack of screening tests, late presentation of disease, lack of effective treatment, as well as the uncertain etiology of pancreatic cancer [2–5]. Cigarette smoking, obesity, pancreatitis, and family history of pancreas cancer are established risk factors [6–14]. In addition, diabetes may be associated with pancreatic cancer risk or it may be an early indicator of disease [15–17].

Many studies have assessed the association between alcohol consumption and pancreatic cancer risk; although the evidence remains inconsistent, it suggests an increased risk associated with heavy drinking [18–30]. Several pooled studies and a meta-analysis of the alcohol—pancreatic cancer association have been conducted. A pooled analysis of 14 cohort studies found a modest increased pancreatic cancer risk with consumption of 30 or more grams of alcohol per day [18]. A meta-analysis of case-control and cohort studies demonstrated an increased risk for pancreatic cancer among persons consuming 3 or more drinks per day [19]. Similarly, a pooled analysis of 10 case-control studies found a significantly increased pancreatic cancer risk associated with 9 or more drinks per day, with no association found for occasional and light—moderate drinkers [20]. Conversely, a pooled analysis of 12 cohort studies observed no association between total alcohol intake and pancreatic cancer risk, however a modest association was observed among men that drank heavily, but not women [21].

Our study investigated the association between alcohol consumption and pancreatic cancer risk with stratification by sex and smoking status.

## Materials and Methods

### Case ascertainment and response rates

Pancreas cancer cases were recruited through the Ontario Pancreas Cancer Study, as previously described [31]. The current analyses included recent cases diagnosed between February 1, 2011 and August 31, 2012. Pancreatic cancer cases were men and women, ≤ 89 years of age, diagnosed with a pathologically-confirmed adenocarcinoma of the pancreas (International Classification of Diseases for Oncology Third Edition (ICD-03, C25.0–25.9)). Confirmation of case diagnosis and permission to contact patients was requested from physicians. Cases were then mailed a study package that included self-administered questionnaires on family history of cancer, epidemiology/lifestyle factors, allergies and food intake. After two weeks, a reminder post card was sent and telephone calls were made another two weeks later to those who had not responded; a second package was sent upon request, plus additional telephone follow-up for non-responders.

Of the 1,095 individuals diagnosed with pancreatic cancer during this time period, 262 (24%) were ineligible (deceased, language barrier, resided outside Ontario), or were unable to be contacted (physician refused, had no address, no physician to contact for consent). Of the 833 that were invited to participate, 195 (23%) were later found to be ineligible (deceased, language restrictions, living outside of Ontario, or older than 89 years). Of the 638 eligible pancreas cancer cases, 345 (54%) completed the questionnaires, 137 (22%) refused, 46 (7%) were unable to be contacted, 30 (5%) provided DNA samples only, and 80 (13%) were non-responders. The final 345 participants were comprised of 308 (89%) cancer cases and 37 (11%) proxy respondents.

### Control ascertainment and response rates

Population-based controls were frequency-matched (1:3) within 5-year age/sex groups to the expected pancreas cancer case distribution. Controls were recruited (during 2011) through random digit dialing methods previously described [31]. Of the 11,629 households telephoned, 1,995 contained an eligible individual and of these, 1,734 (87%) persons agreed to participate. These controls were mailed a study package from the Ontario Cancer Risk Factor Study. Reminder post cards were sent two weeks later and non-responders were followed up at four weeks and eight weeks with a telephone call; at ten weeks they were re-mailed the package. Questionnaires were completed by 1,285 controls (74% response rate).

### Alcohol variables

Both cases and controls completed questionnaires, which included: medical history, medication use, dietary intake, physical activity, body weight, reproductive history, chemical exposures, family history of pancreas cancer, alcohol intake, and smoking. Alcohol consumption two years ago (recent) was asked about separately for beer, white wine, red wine, and liquor. Alcohol consumption frequency options were: never or less than once per month, less than once per week, at least once per week (1–6 drinks/week), at least once per day (7–21 drinks/week), or more than 3 per day (> 21 drinks/week). A conservative value was assigned to each of these frequency options for each type of alcohol such that: drinking never or less than once per month was 0, less than once per week was 0.4, at least once per week was 1, at least once per day was 7, and more than 3 per day was 21. These values were then summed across the four types of alcohols for each individual to obtain a measure of total alcohol consumption (two years ago), which was then categorized into groups (0 –< 1 drink/week reference category, 1–3 drinks/week, 4–20 drinks/week, and ≥ 21 drinks/week). The highest level of alcohol consumption was set at ≥ 21 drinks/week because evidence in the literature showed associations at this ‘heavy’ threshold amount [19].

In addition to consumption frequency, subjects were also categorized based on the type of alcoholic beverages. If total alcohol intake was never or less than once per month, persons were considered never drinkers for each type of alcoholic beverage. Next, those who drank only one particular alcoholic beverage were considered drinkers of only that particular alcoholic beverage. Mixed drinkers were persons who drank two or more types of alcoholic beverages. This produced the categories: never any, beer only, wine only, liquor only, and mixed drinker. Subjects with missing values for alcohol intake were considered never drinkers (less than 2% of persons had missing values), which was assumed for both the alcohol consumption and alcohol type variables.

### Ethical approval

The study protocol(s) and consent procedure(s) were approved by the Mount Sinai Hospital Research Ethics Board and the University of Toronto Research Ethics Board. For questionnaire data, consent was implied as the subject completed the self-administered questionnaires and returned them by mail. Each participant had the freedom to decline or withdraw from this study at any given point in time.

### Statistical analysis

All analyses were conducted using SAS 9.2 (SAS Institute Inc.). The distribution of cases and controls for established risk factors were described. Confounding was assessed for the association between alcohol consumption and pancreatic cancer risk by adding each potential confounder (sex, age, body mass index (based on weight one year prior to questionnaire completion), type 2 diabetes, pancreatitis, family history of pancreas cancer, smoking status (non-smoker, current, former)) to the age-adjusted logistic regression model and comparing the change in the AOR to the crude model containing only age-group and the alcohol consumption variable. A change in the AOR of ≥ 15% suggested the variable was a confounder and would remain in the final multivariate models [32]. This assessment revealed that none of the covariates were confounders; thus, the most parsimonious model was that controlled for age-group only. The association between alcohol consumption and pancreatic cancer risk was stratified by sex and smoking status to investigate effect modification.

## Results

The distribution of subject characteristics and established risk factors for cases and controls are shown in Table 1. As expected (and previously shown [31]), the following were associated with a significant increased risk of pancreatic cancer: diabetes, family history of pancreas cancer, pancreatitis, and smoking.

**Table 1:** Description of study participants, including age-adjusted odds ratio (AOR) estimates for pancreatic cancer risk factors.

	Cases (N = 345)		Controls (N = 1285)		AOR
Risk Factor	N	%	N	%	(95% CI)
**Body Mass Index (kg/m** ^**2**^ **)** ^**a**^					
Normal/underweight (< 25.0)	119	35	412	32	1.0
Overweight (25.0 – 30.0)	110	33	522	41	0.7 (0.6 – 1.0)
Obese (> 30.0)	107	32	343	27	1.1 (0.8 – 1.5)
**Diabetes** ^**b**^					
No	270	78	1113	87	1.0
Yes	74	22	162	13	1.7 (1.3 – 2.4)
**Family history of pancreas cancer** ^**c**^					
No	298	91	1168	96	1.0
Yes	29	1	49	4	2.4 (1.5 – 3.8)
**Pancreatitis**					
No	325	95	1261	99	1.0
Yes	17	5	15	1	4.4 (2.2 – 9.1)
**Smoking**					
Never	135	40	581	45	1.0
Current	60	18	157	12	1.9 (1.3 – 2.7)
Former	145	43	544	42	1.1 (0.9 – 1.4)
**Sex**					
Male	175	51	680	53	—
Female	170	49	605	47	
**Age Group (years)** ^**d**^					
<60	86	25	450	35	—
60 – 64	74	21	288	22	
65 – 69	65	19	221	17	
≥70	120	35	326	25	

**Abbreviations:** AOR: age-adjusted odds ratio, CI: confidence interval

Numbers may not add to total due to missing values

^a^ One year before diagnosis/questionnaire completion

^b^ Prior to one year before diagnosis/questionnaire completion; Type 2 diabetes

^c^ First degree relative

^d^ Age at pancreatic cancer diagnosis for cases and age at questionnaire completion for controls

Table 2 shows the AORs for the association between total alcohol consumption, alcohol type, and pancreatic cancer risk. Heavy alcohol consumption (≥ 21 drinks/week) was associated with an increased risk of pancreatic cancer (AOR = 1.35, 95% CI: 0.81, 2.27), however, this association was not statistically significant. No statistically significant increased risk was found for any type of alcoholic beverage.

**Table 2:** Age-adjusted odds ratio (AOR) estimates for total alcohol consumption and alcohol type.

	Cases (n = 345)		Controls (N = 1285)		AOR
	N	%	N	%	(95% CI)
**Alcohol Consumption** ^**a**^					
0 - <1 drinks/week	167	48	573	45	1.00
1–3 drinks/week	82	24	372	29	0.78 (0.58 – 1.05)
4–20 drinks/week	73	21	283	22	0.86 (0.63 – 1.17)
≥ 21 drinks/week	23	7	57	4	1.35 (0.81 – 2.27)
**Alcohol Type** ^**a**^					
Never any^b^	133	39	399	31	1.00
Beer only	29	8	87	7	1.09 (0.68 – 1.75)
Wine only	27	8	120	9	0.66 (0.42 – 1.05)
Liquor only	11	3	33	3	0.97 (0.48 – 1.99)
Mixed (beer/wine/liquor)	145	42	646	50	0.67 (0.51 – 0.88)

**Abbreviations:** AOR: age-adjusted odds ratio (assessment of covariates showed no confounders), CI: confidence interval

^a^ 2 years prior to diagnosis/questionnaire completion

^b^ Beer, red wine, white wine, and liquor consumption was never or less than once per month

The association between alcohol consumption and pancreatic cancer risk stratified by sex is shown in Table 3. There was no significant difference in AORs observed between males and females (*P* = 0.27). The dataset was also stratified by smoking status as shown in Table 4. Among current smokers, an increase in pancreatic cancer risk was associated with alcohol consumption (light, moderate, and heavy); this was statistically significant for 1–3 alcoholic drinks/week (AOR = 2.31, 95% CI: 1.03, 5.20) and ≥ 21 drinks/week (AOR = 4.04, 95% CI: 1.58, 10.37). This statistically significant association between pancreatic cancer risk and heavy drinking was not observed among non-smokers (*P*_interaction_ = 0.008). It is important to note the large confidence intervals around the heavy drinker AOR within the non-smoking group. Upon further exploration, it was observed that heavy alcohol drinkers were more likely to be heavy smokers (data not shown). Therefore, to be conservative, we forced the smoking pack-years variable into the current smoker model; the AORs remained similar to the model without pack-years with significant AORs for alcohol intake in the range of a doubling of pancreatic cancer risk (the most parsimonious model is presented here).

**Table 3:** Association between alcohol consumption and pancreatic cancer risk stratified by sex.

	Males			Females			
Alcohol Consumption (drinks/week)^a^	Cases	Controls	AOR (95% CI)	Cases	Controls	AOR (95% CI)	P-value for
	N (%)	N (%)		N (%)	N (%)		Interaction
0 – <1	73 (42)	235 (35)	1.00	94 (55)	338 (56)	1.00	0.27
1 – 3	45 (26)	206 (30)	0.73 (0.48 – 1.10)	37 (22)	166 (27)	0.82 (0.54 – 1.26)	
4 – 20	42 (24)	197 (29)	0.68 (0.44 – 1.04)	31 (18)	86 (14)	1.23 (0.76 – 1.98)	
≥ 21	15 (9)	42 (6)	1.14 (0.59 – 2.17)	8 (5)	15 (2)	1.92 (0.78 – 4.75)	

**Abbreviations:** AOR: age-adjusted odds ratio (assessment of covariates showed no confounders), CI: confidence interval

^a^ 2 years prior to diagnosis/questionnaire completion

**Table 4:** Association between alcohol consumption and pancreatic cancer risk stratified by smoking status.

	Never Smokers			Current Smokers			Former Smokers			
Alcohol Consumption (drinks/week)^a^	Cases	Controls	AOR (95% CI)	Cases	Controls	AOR (95% CI)	Cases	Control	AOR (95% CI)	P-value for Interaction
	N (%)	N (%)		N (%)	N (%)		N (%)	N (%)		
0 – <1	80 (59)	301 (52)	1.00	18 (30)	82 (52)	1.00	65 (45)	187 (34)	1.00	0.008
1 – 3	33 (24)	172 (30)	0.78 (0.50 – 1.24)	15 (25)	30 (19)	2.31 (1.03 – 5.20)	34 (23)	170 (31)	0.58 (0.36 – 0.93)	
4 – 20	19 (14)	101 (17)	0.66 (0.38 – 1.14)	15 (25)	32 (20)	2.16 (0.96 – 4.87)	38 (26)	150 (28)	0.69 (0.44 – 1.10)	
≥ 21	3 (2)	7 (1)	2.01 (0.50 – 8.18)	12 (20)	13 (8)	4.04 (1.58 – 10.37)	8 (6)	37 (7)	0.55 (0.24 – 1.24)	

**Abbreviations:** AOR: age-adjusted odds ratio (assessment of covariates identified no confounders), CI: confidence interval

Numbers may not add to total due to missing values

^a^ 2 years prior to diagnosis/questionnaire completion

## Discussion

Overall, we found that alcohol consumption was not associated with pancreatic cancer risk; however, a non-significant increased risk was observed with heavy drinking. Furthermore, smoking modified the association between alcohol and pancreatic cancer risk, such that among current smokers, consuming alcohol (light, moderate, and heavy) was associated with a more than two-fold increased pancreatic cancer risk, which was statistically significant among heavy drinkers.

Consistent with previous prospective cohort studies [23, 24], a case-control study [25], and a pooled cohort study [21], heavy alcohol consumption was not significantly associated with pancreatic cancer risk. However, we also observed a non-statistically significant increased risk of pancreatic cancer with heavy alcohol consumption, which is consistent with pooled cohort and case-control studies [18, 20], a meta-analysis [19], and prospective cohort studies [27–30] showing a significant positive association between heavy alcohol consumption and pancreatic cancer risk. It is possible our small sample size did not provide adequate power to detect a statistically significant association.

Consistent with some studies [18, 23], no associations were found for individual alcohol types (beer, white wine, red wine, liquor), possibly due to the low frequencies observed within these groups. However, other prospective cohort studies reported positive associations for consumption of liquor as compared to beer and wine [24, 28].

Possible effect modification by sex is inconsistent in the literature. The current study found that sex did not significantly modify the association between alcohol consumption and pancreatic cancer risk, which is similar to that found in a pooled cohort study and meta-analysis [18, 19]. However another pooled cohort study [21] and population-based case-control study [33] found an increased risk of pancreatic cancer with alcohol consumption in men, but not in women.

Previous population-based case-control studies [33], pooled case-control and pooled cohort studies [6, 18, 21], and a prospective cohort study [28] reported that smoking status was not an effect modifier of the association between alcohol consumption and pancreatic cancer risk. However, four of these studies found a non-significant increase in pancreatic cancer risk with heavy alcohol consumption among current smokers compared to non-smokers [6, 18, 28, 33]. These findings are similar to our study, which found a strong and statistically significant association between alcohol consumption and pancreatic cancer risk among current smokers only. The inconsistencies could possibly be attributed to sample size constraints and differences in adjustment for covariates. After stratification by smoking status, our data was quite sparse; therefore our findings must be interpreted with caution until replicated in larger studies.

Although ethanol and its metabolite, acetaldehyde, are considered carcinogens, the role of alcohol consumption in the development of pancreatic cancer remains unclear [34]. Many possible biologic mechanisms have been proposed regarding how alcohol may lead to pancreas damage and finally pancreatic cancer, such as: oxidative stress, cellular damage, upregulated NADH/cytochrome P450 enzymes, and free radical formation [22, 34–39].

Smoking causes DNA damage [40, 41] and a recent review suggests that heavy alcohol consumption and smoking may have overlapping effects during pancreatic carcinogenesis [22]. For example, chronic alcohol consumption increases cytochrome P450 activity, increasing cigarette smoke metabolites that promote carcinogenesis [22, 36, 42–44]. It has also been proposed that nicotine may increase ethanol metabolism in the pancreas, amplifying damage [35].

The strengths of this study include: i) valid case selection based on pathological confirmation of pancreatic cancer cases, ii) a short lag time between diagnosis and questionnaire completion (approximately 3 months) as a result of electronic pathology reporting to the cancer registry, and iii) population-based cases and controls. However, all studies have limitations. Although temporality was established as exposures referred to two years prior to diagnosis, case-control studies are subject to possible information bias as it is difficult to obtain accurate measurements of past exposures. Research also shows that heavy drinkers are more likely to have difficulties with memory recall [45]. In addition, this case-control study had a relatively small sample size limiting the power to detect associations, especially in subgroups. Of course, the possibility that our findings are due to chance cannot be ruled out.

## Conclusions

The current study somewhat supports the literature suggesting that heavy alcohol intake may increase one’s risk of developing pancreatic cancer. In addition, among current smokers, even light drinking may be associated with a doubling of pancreatic cancer risk. Due to sparse data after stratifying by smoking status, it is important that these findings be replicated in larger future studies.
